# Recursive Partitioning vs Computerized Adaptive Testing to Reduce the Burden of Health Assessments in Cleft Lip and/or Palate: Comparative Simulation Study

**DOI:** 10.2196/26412

**Published:** 2021-07-30

**Authors:** Conrad J Harrison, Chris J Sidey-Gibbons, Anne F Klassen, Karen W Y Wong Riff, Dominic Furniss, Marc C Swan, Jeremy N Rodrigues

**Affiliations:** 1 Nuffield Department of Orthopaedics, Rheumatology and Musculoskeletal Sciences University of Oxford Oxford United Kingdom; 2 MD Anderson Center for INSPiRED Cancer Care University of Texas Houston, TX United States; 3 Department of Pediatrics McMaster University Hamilton, ON Canada; 4 Department of Plastic and Reconstructive Surgery Hospital for Sick Children Toronto, ON Canada; 5 Spires Cleft Centre John Radcliffe Hospital Oxford United Kingdom; 6 Warwick Clinical Trials Unit University of Warwick Coventry United Kingdom; 7 Department of Plastic Surgery Stoke Mandeville Hospital Buckinghamshire Healthcare NHS Trust Aylesbury United Kingdom

**Keywords:** cleft Lip, cleft palate, patient-reported outcome measures, outcome assessment, CLEFT-Q, computerized adaptive test, machine learning, decision tree, regression tree

## Abstract

**Background:**

Computerized adaptive testing (CAT) has been shown to deliver short, accurate, and personalized versions of the CLEFT-Q patient-reported outcome measure for children and young adults born with a cleft lip and/or palate. Decision trees may integrate clinician-reported data (eg, age, gender, cleft type, and planned treatments) to make these assessments even shorter and more accurate.

**Objective:**

We aimed to create decision tree models incorporating clinician-reported data into adaptive CLEFT-Q assessments and compare their accuracy to traditional CAT models.

**Methods:**

We used relevant clinician-reported data and patient-reported item responses from the CLEFT-Q field test to train and test decision tree models using recursive partitioning. We compared the prediction accuracy of decision trees to CAT assessments of similar length. Participant scores from the full-length questionnaire were used as ground truth. Accuracy was assessed through Pearson’s correlation coefficient of predicted and ground truth scores, mean absolute error, root mean squared error, and a two-tailed Wilcoxon signed-rank test comparing squared error.

**Results:**

Decision trees demonstrated poorer accuracy than CAT comparators and generally made data splits based on item responses rather than clinician-reported data.

**Conclusions:**

When predicting CLEFT-Q scores, individual item responses are generally more informative than clinician-reported data. Decision trees that make binary splits are at risk of underfitting polytomous patient-reported outcome measure data and demonstrated poorer performance than CATs in this study.

## Introduction

### Computerized Adaptive Testing

Computerized adaptive testing (CAT) describes the use of algorithms to shorten and personalize questionnaires by selectively administering only the most relevant items to an individual based on the responses they have already given during that assessment [1]. The item that is expected to be most relevant (ie, most informative) in a general population is picked first [2,3]. Once the individual has responded, an algorithm predicts the person’s score and selects the most relevant item to ask next, based on the predicted score and an item selection criterion. This continues iteratively until a stopping rule is met.

There are some limitations in this approach to personalized assessments. Firstly, this method is only possible in patient-reported outcome measures (PROMs) that fit item response theory (IRT) or Rasch models, and not those that only meet the structural requirements of classical test theory [1,4]. Secondly, an individual’s score must be calculated after each response to select the next item, which can be computationally demanding in situations where the assessment has many different items to choose from (ie, item-banking) and may cause a time delay between each item. Thirdly, CAT assessments do not directly incorporate clinician-reported variables. These variables, which can be automatically captured from a person’s electronic health record, may be very informative and can potentially improve the efficiency and accuracy of personalized assessments.

### Recursive Partitioning

Recursive partitioning is a form of machine learning that involves iteratively splitting labeled data sets into subgroups to minimize the within-subgroup variance of an outcome, such as a PROM score [5]. Recent studies have explored the use of personalized health assessments based on decision trees constructed with similar techniques [6-8]. These trees split respondents into subgroups based on their responses to individual items.

The use of decision trees in personalized health assessment may be appealing because they are not restricted by IRT model requirements, and trees are developed *a priori* (ie, they do not need to calculate a person’s score between each item), attenuating potential lag time [9]. It may also be possible to use recursive partitioning to split data based on clinical variables other than item responses, meaning that, unlike traditional CAT assessments, decision trees could automatically incorporate clinical information known to predict a person’s score. For example, the frequency of inhaler prescriptions could guide item selection in an asthma assessment, and step count could be incorporated into an assessment of mobility. However, many PROMs comprise polytomous response options, which may not be handled well by binary decision nodes.

To our knowledge, the potential for recursive partitioning to improve the accuracy and efficiency of CAT by incorporating clinician-reported variables into patient-reported assessments has not been explored prior to this study.

### The CLEFT-Q

A cleft lip and/or palate (a split in the upper lip, gum, and/or roof of the mouth) is one of the most common birth anomalies. It affects 1 in 700 births and can impact various health domains, including appearance, speech, and psychosocial development [10].

In this study, we developed CAT models and decision trees for scales of the CLEFT-Q. The CLEFT-Q is a PROM intended for use in children and young adults born with a cleft lip and/or palate [11]. It includes 12 independent scales that have met Rasch model requirements, measuring the perceived appearance of the respondent’s face, nose, nostrils, lips, cleft lip scar, jaw, and teeth, as part of an “appearance” domain; speech function, as part of a “facial function” domain; and speech distress, psychological function, school function, and social function, as part of a “quality of life” domain. Differences in CLEFT-Q scale scores have been associated with the overall patient-reported perception of appearance [11] and several clinical variables, including cleft type [12], use of psychological therapy, [11] and clinician-reported plans for future surgery [13].

### Hypothesis

We tested the null hypothesis that adaptive assessments incorporating clinical variables and item responses (created using recursive partitioning) would predict CLEFT-Q scale scores with similar accuracy to CAT assessments of a comparable length.

## Methods

### Software

We conducted our analysis using R (version 4.0.0) with the following packages: dplyr (version 1.0.0), foreign (version 0.8-80), mirt (version 1.32.1), mirtCAT (version 1.9.3), partykit (version 1.2-8), rpart (version 4.1-15), rpart.plot (version 3.0.8), stringr (version 1.4.0) and ggplot2 (version 3.3.2).

### Study Participants

We used data from the CLEFT-Q field test to construct and test our models. This prospective study recruited 2434 participants across 12 countries born with a cleft lip and/or palate, aged 8 to 29 years, from October 2014 to November 2016. Responses to all items in relevant full-length CLEFT-Q scales were collected in addition to clinical information. The CLEFT-Q field test study and its participants have been described in detail elsewhere [11,14].

These participants’ complete response sets were used to develop and test CAT and decision tree models. Participants with a cleft palate only were excluded from analyses relating to the lip and cleft lip scar scales. Patients without a cleft palate were excluded from analyses relating to the speech function and speech distress scales. This reflects populations expected to use these assessments in clinical practice.

### Missing Data

Some functions in the mirt package require complete response sets [15]. Where applicable, we excluded participants with incomplete response sets listwise. Otherwise, missing data were handled with pairwise exclusion. In the CLEFT-Q field test, only participants aged 12 to 29 years completed the jaw scale, only participants born with a cleft lip completed the cleft lip scar scale, and only participants attending school completed the school scale [11]. There were no other apparent patterns in missing data. We provide descriptive statistics, including a summary of missing data in a demographic table (see Multimedia Appendix 1).

### Ground Truth

**Figure 1:**
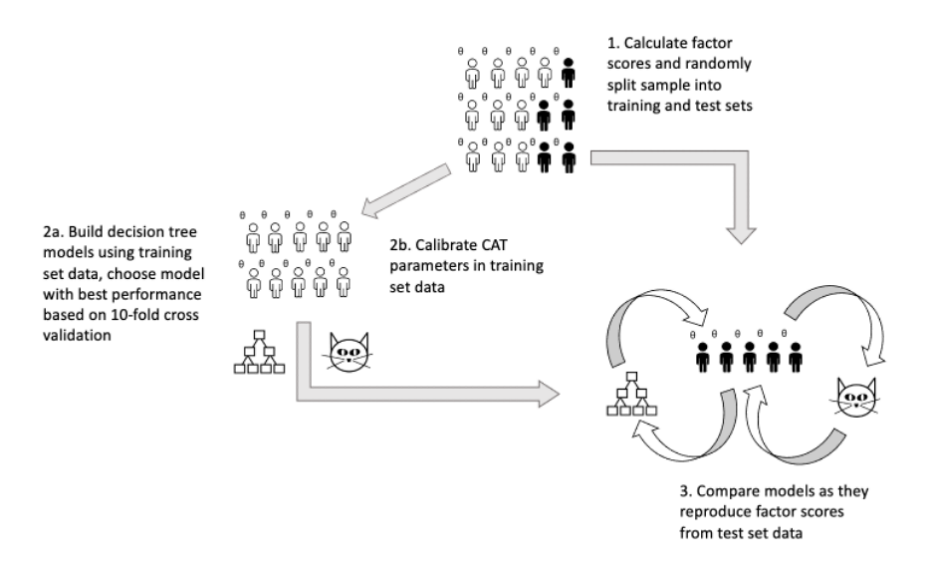
Schematic describing the steps of the CAT vs recursive partitioning experiment. CAT: computerized adaptive test.

Rasch models were developed for each scale using all item response sets from the whole sample. Model parameters were estimated using a fixed quadrature expectation-maximization (EM) approach [15]. For each participant, expected *a posteriori* (EAP) factor scores were calculated for each scale using full-length scale responses and the respective Rasch model parameters. These scores are presented as person-location logits and represent ground truth in these experiments (Figure 1).

### Data Splitting

Participants with complete response sets were selected from the study sample and randomly split into training and test sets in a 2:1 ratio for each scale. Next, training samples were used to create new Rasch models using the EM algorithm, and CAT models were generated using the parameters of these training sample Rasch models. Finally, decision trees were built through recursive partitioning in 10-fold cross-validations of the same training samples.

### Sample Size

All of our training data sets approached or exceeded a sample size of 1000 (see Multimedia Appendix 1), which is likely to be sufficient for accurate regression tree development based on previous simulation studies [16]. For CAT, sample sizes of 250 or more can provide definitive Rasch model calibrations with over 99% confidence [17].

### Item Responses

The data sets used to train our decision tree models included responses to all items in each CLEFT-Q scale. Items in the speech distress and speech function scale had 3 response options, and all other items had 4 response options. In addition to these items, the CLEFT-Q field test included 7 questions that asked respondents about the overall appearance of their face, nose, nostrils, lips, cleft lip scar, teeth, and jaws. There were 4 response options to these items. These responses were included in the relevant decision tree training data set, meaning, for example, that the decision tree nose assessment was able to administer an additional item that asked about overall nose appearance. The CAT model could not use this item as it was not part of the Rasch-validated scale.

### Clinical Variables

In addition to item responses, the training data sets included several clinically relevant variables, for example, age, gender, cleft type, planned future treatments, and patient-perceived appearance scores. The variables included in each model are listed in Multimedia Appendix 2.

### Decision Tree Training

Regression trees could make binary splits based on item responses or any of the included clinical variables. We grew trees to a prespecified maximum depth corresponding to the number of items permitted by their CAT comparator. Tree growth was not limited by observations per node or by a prespecified complexity parameter. Branches that did not improve model fit were removed retrospectively through cost-complexity pruning. For this, we used training data to choose complexity parameters that minimized prediction error in a 10-fold cross-validation.

In other words, for each scale, many different decision trees were created from and evaluated in different folds of the training data set. The model that predicted scores with the least error in the training data set cross-validation was selected for final assessment in the test data set.

### Computerized Adaptive Tests

The CATs were programmed to select items using a minimum expected posterior variance item selection criterion [18]. Finally, participants were scored using an EAP approach [15].

### Assessment Length

We aimed to compare the accuracy of CAT assessments and decision trees that had a similar number of items. For this reason, we chose fixed-length stopping rules to limit the number of items administered in the CAT models. The CAT algorithms would continue to administer items until the fixed-length stopping rule was met, and they were not limited by time, standard error of measurement, or any other stopping criterion. It was not possible to predetermine decision tree assessment lengths. This is because splits could be made based on clinical variables, and some branches were pruned, creating inconsistent assessment lengths. Instead, tree growth was limited by depth, and the number of items required to reach each terminal node was recorded. If a decision tree made a split based on overall patient-reported appearance (ie, the additional question posed to CLEFT-Q field test participants, not included in the scale), we counted this as an item. Splits based on clinician-reported variables were not counted as items. For each scale, we compared models at 2 maximum assessment lengths, which were approximately 75% and 50% of the length of the whole scale.

### Comparison Methods

For each respondent in the test data set, person-location logits were predicted by decision trees and their CAT comparators in Monte Carlo simulations. For each model, we recorded the mean number of items administered, which items and clinical variables were used to make predictions, the Pearson’s correlation coefficient of predictions and ground truth, and the mean absolute error (MAE) of the predictions with respect to ground truth. Additionally, we calculated the root mean squared error of predictions, which is typically reported in CAT simulations and tends to penalize large errors that are potentially important in this context [19].

Squared CAT errors and squared decision tree errors were compared using a two-sided Wilcoxon signed-rank test.

## Results

Assessment length was generally similar between decision trees and CAT assessments (see Multimedia Appendix 1). Notable exceptions to this were the nose assessments limited to 9 items (mean of 7.32 items in decision tree assessments vs 9.00 items in CAT assessments), and the speech function assessments were limited to 9 items (mean of 7.01 items in decision tree assessments vs 9.00 items in CAT assessments).

For most comparisons, the squared error was significantly higher (*P*<.001) in decision tree predictions than in predictions made by CAT assessments at comparable or slightly shorter mean assessment lengths. The poor accuracy of the decision tree models compared to CAT was also captured by correlation coefficients and MAE values (see Multimedia Appendix 1).

While information about age, gender, cleft type, laterality, and patient-reported overall appearance scores were used by some of the deeper decision trees, these algorithms tended to make splits based on CLEFT-Q scale item responses preferentially (ie, other variables were either not used, used at deeper levels, or removed through pruning). An exception to this was the nose assessment decision tree, which made its first split based on the patient-reported overall appearance of the nose.

## Discussion

### Principal Findings

**Figure 2:**
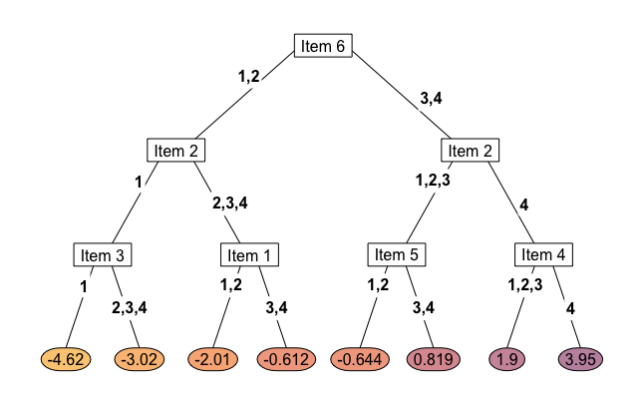
Dendrogram of the three-item decision tree for the nostrils scale. Nodes are represented by rectangular boxes. Branches are labelled with splitting criteria . Leaves (terminal nodes) are represented by coloured ovals and are labelled with the mean person location logit for training dataset respondents falling into that subgroup.

This study has shown that it is technically possible for decision trees built through recursive partitioning to use clinician-reported data to reduce the patient-reported assessment burden in a range of cleft-related health domains. However, this approach has demonstrated little clinical value with regard to the CLEFT-Q. Decision trees preferentially made splits based on patient-reported item responses and not clinician-reported data. One way to interpret this in real-world terms is that the clinical variables used in this study are less important than almost any individual CLEFT-Q item response for measuring a person’s cleft-related health state (Figure 2). However, this finding is not necessarily generalizable. Differences in cleft phenotype may have a relatively mild impact on health constructs measured by the CLEFT-Q. Clinician-reported variables may be more salient in other health domains. For example, “history of spinal cord injury” may be a powerful predictor of the physical functioning score.

**Figure 3:**
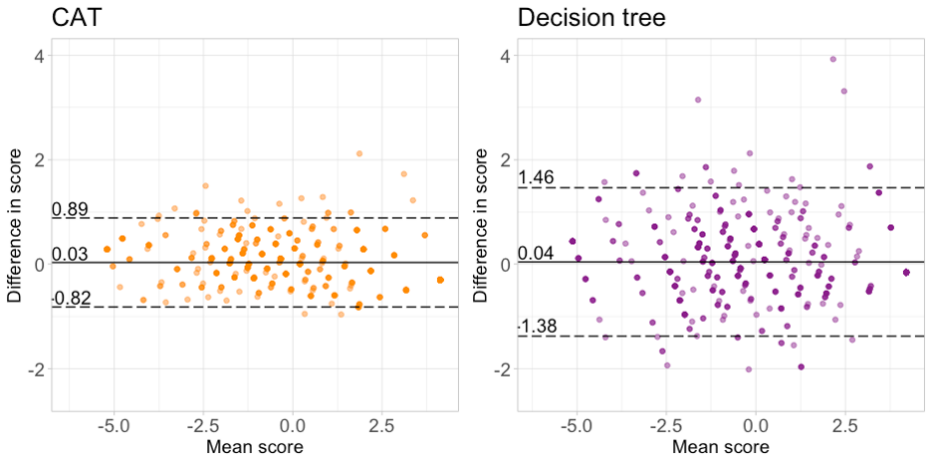
Bland-Altman plots comparing the accuracy of personalized CLEFT-Q lip appearance assessments. The left panel demonstrates the results for a computerized adaptive test with a mean length of 5.00 items. The right panel demonstrates the results for a decision tree with a mean length of 4.95 items.

Our second finding was that decision trees produced more error than CAT assessments of a comparable length (Figure 3; Multimedia Appendix 1). CLEFT-Q scales are short (6 to 12 items), which means CAT item selection is relatively computationally undemanding in this specific case. Any benefits in lag time gained by the recursive partitioning approach are unlikely to outweigh this loss of accuracy. Therefore, we would not advocate the use of binary decision trees for adaptive CLEFT-Q assessments.

In this study, we used recursive partitioning for tree construction, which is limited to binary data splits (ie, individuals can be categorized into only 2 subgroups after providing an item response; Figure 2). There are 3 or 4 possible responses to each CLEFT-Q item, and therefore the CAT models can categorize a respondent in 3 or 4 ways each time they provide an item response. For polytomous PROM scales, there are far fewer attainable scores in a binary decision tree than in a CAT assessment of equivalent length. For example, in the speech function assessment at a 6-item limit, there were 85 unique scores achieved by our CAT models compared to 45 in the decision tree group. Our decision trees, therefore, underfit the test data to an extent.

A way to overcome this in future work could be through constructing trees with nonbinary splits, for example, by treating all variables as categorical and using chi-square automatic interaction detection (CHAID) [20,21]. This technique could have a higher risk of overfitting, although this might be mitigated through split rules and by merging similar branches [6]. Future work is needed to test whether CHAID could create more efficient, more accurate, adaptive assessments by incorporating non-PROM data at no extra burden to the assessment taker.

A limitation of this study is that some comparisons were made on assessments of unequal mean length; for example, the nose assessment was limited to 9 items, and the speech function assessment was limited to 9 items. In these cases, it is difficult to conclude the relative accuracy of CAT and decision trees, as these models used different quantities of patient-reported information to make their measurements. In addition, decision trees tended to pose fewer questions than their CAT comparators due to the pruning process and the use of clinical variables. However, even with this limitation in mind, our findings support the use of CAT over binary trees for personalized CLEFT-Q assessments in clinical practice.

### Conclusions

Even with knowledge of clinician-reported variables, the decision tree models described in this study achieve less accurate CLEFT-Q score estimates than CATs of similar length. Decision trees with binary splits are at risk of underfitting polytomous PROM scale data. Future work could focus on the application of decision trees with nonbinary splits to this context.

## Appendix

Multimedia Appendix 1 Supplement 1 - results.

Multimedia Appendix 2 Supplement 2.

## Abbreviations

CAT: computerized adaptive test
CHAID: chi-square automatic interaction detection
EAP: expected a posteriori
EM: expectation-maximization
IRT: item response theory
MAE: mean absolute error
PROM: patient-reported outcome measure
