# Supplementary material for: Recursive Partitioning vs Computerized Adaptive Testing to Reduce the Burden of Health Assessments in Cleft Lip and/or Palate: Comparative Simulation Study
Source: J Med Internet Res. 2021 Jul 30;23(7):e26412. doi: 10.2196/26412 (PMC8367147; doi:10.2196/26412)
Supplement: Multimedia Appendix 2 [file jmir_v23i7e26412_app2.docx]

Supplement 2: Variables included in decision tree training data

Decision trees were trained on datasets that included all item responses and a number of clinically relevant variables.

For the lip scale models this included: age, gender, cleft type (cleft lip, cleft lip and alveolus or cleft lip, alveolus and palate), laterality (unilateral or bilateral), unilateral lip type (complete, incomplete or lesser form), bilateral lip type (complete, incomplete, lesser form or asymmetric), planned primary lip repair (yes or no), planned lip scar revision (yes or no), overall lip appearance reported by the participant on a four-point ordinal scale, and nine lip scale items reported on a four-point ordinal scale.

For the cleft lip scar scale models this included: age, gender, cleft type (cleft lip, cleft lip and alveolus or cleft lip, alveolus and palate), laterality (unilateral or bilateral), unilateral lip type (complete, incomplete or lesser form), bilateral lip type (complete, incomplete, lesser form or asymmetric), planned lip scar revision (yes or no), overall cleft lip scar appearance reported by the participant on a four-point ordinal scale, and seven cleft lip scar scale items reported on a four-point ordinal scale.

For the teeth scale models this included: age, gender, cleft type (cleft lip, cleft palate, cleft lip and alveolus, or cleft lip, alveolus and palate), laterality (unilateral or bilateral), planned alveolar bone grafting (yes or no), planned orthodontic treatment (yes or no), overall teeth appearance reported by the participant on a four-point ordinal scale, and eight teeth scale items reported on a four-point ordinal scale.

For the jaw scale models this included: age, gender, cleft type (cleft lip, cleft palate, cleft lip and alveolus, or cleft lip, alveolus and palate), laterality (unilateral or bilateral), planned orthognathic surgery (yes or no), overall jaw appearance reported by the participant on a four-point ordinal scale, and seven jaw scale items reported on a four-point ordinal scale.

For the nose scale models this included: age, gender, cleft type (cleft lip, cleft palate, cleft lip and alveolus or cleft lip, alveolus and palate), laterality (unilateral or bilateral), unilateral lip type (complete, incomplete or lesser form), bilateral lip type (complete, incomplete, lesser form or asymmetric), planned rhinoplasty (yes or no), overall nose appearance reported by the participant on a four-point ordinal scale, and 12 nose scale items reported on a four-point ordinal scale.

For the nostrils scale models this included: age, gender, cleft type (cleft lip, cleft palate, cleft lip and alveolus or cleft lip, alveolus and palate), laterality (unilateral or bilateral), unilateral lip type (complete, incomplete or lesser form), bilateral lip type (complete, incomplete, lesser form or asymmetric), planned rhinoplasty (yes or no), overall nostril appearance reported by the participant on a four-point ordinal scale, and six nostril scale items reported on a four-point ordinal scale.

For the face scale models this included: age, gender, cleft type (cleft lip, cleft palate, cleft lip and alveolus or cleft lip, alveolus and palate), laterality (unilateral or bilateral), unilateral lip type (complete, incomplete or lesser form), bilateral lip type (complete, incomplete, lesser form or asymmetric), overall face appearance reported by the participant on a four-point ordinal scale, and nine face scale items reported on a four-point ordinal scale.

For the psychological function scale models this included: age, gender, cleft type (cleft lip, cleft palate, cleft lip and alveolus or cleft lip, alveolus and palate), laterality (unilateral or bilateral), planned psychological therapy, and ten psychological function scale items reported on a four-point ordinal scale.

For the social function scale models this included: age, gender, cleft type (cleft lip, cleft palate, cleft lip and alveolus or cleft lip, alveolus and palate), laterality (unilateral or bilateral), and ten social function scale items reported on a four-point ordinal scale.

For the school function scale models this included: age, gender, cleft type (cleft lip, cleft palate, cleft lip and alveolus or cleft lip, alveolus and palate), laterality (unilateral or bilateral), and ten school function scale items reported on a four-point ordinal scale.

For the speech distress scale models this included: age, gender, cleft type (cleft palate or cleft lip, alveolus and palate), clinician reported current speech problem (none, mild, moderate, or severe), planned primary palate repair (yes or no), planned speech surgery (yes or no), planned palatal fistula repair (yes or no), planned speech therapy (yes or no) and ten speech distress scale items reported on a three-point ordinal scale.

For the speech function scale models this included: age, gender, cleft type (cleft palate or cleft lip, alveolus and palate), clinician reported current speech problem (none, mild, moderate, or severe), planned primary palate repair (yes or no), planned speech surgery (yes or no), planned palatal fistula repair (yes or no), planned speech therapy (yes or no) and 12 speech function scale items reported on a three-point ordinal scale.
